# Food bank operations: review of operation research methods and challenges during COVID-19

**DOI:** 10.1186/s12889-023-16269-4

**Published:** 2023-09-14

**Authors:** Aida Esmaeilidouki, Mohana Rambe, Amir Ardestani-Jaafari, Eric Li, Barb Marcolin

**Affiliations:** 1 School of Engineering, University of British Columbia, Kelowna, Canada; 2 Faculty of Management, University of British Columbia, Kelowna, Canada

**Keywords:** Food bank operations, Operations research, COVID-19, Systematic review

## Abstract

Food banks have played a crucial role in mitigating food insecurity in affluent countries for over four decades. Throughout the years, academics have researched food banks for a variety of operational problems, resulting in several research papers on the topic. However, despite significant academic interest, the operational challenges and optimization of food bank operations remain under-researched. This study aims to conduct a systematic literature review on food bank operations and provide evidence-based recommendations for addressing prevalent challenges, and provide decision-makers with practical recommendations. In addition, this investigation seeks to investigate the impact of the COVID-19 pandemic on food bank operations. We conducted a comprehensive analysis of academic publications on food bank operations using the Preferred Reporting Items for Systematic Reviews and Meta-Analyses (PRISMA) in order to get a deeper comprehension of the problems confronting food bank operations. Using a keyword search strategy with the logical operators “AND” and “OR,” two search methods were utilized to identify relevant articles on food bank operations management, supply chain, distribution, and production in our first search. In our second search, we discovered articles in the “Operations Research & Management Science” (OR &MS) category of Web of Science containing food bank-related keywords such as food charity, food donation, and food aid. The database searches yielded 246 hits, and the article content was scanned to eliminate irrelevant articles by removing non-English articles and duplicated studies, leaving 55 articles for further examination. Our extensive examination of Operations Research (OR) methodologies reveals that Mixed-Integer Linear Programming (MILP) models are the most commonly used methodology, followed by Linear Program (LP), Dynamic Program (DP), and Data Envelopment Analysis (DEA) techniques. The key findings of this study emphasize the operational challenges food banks encountered during and after the COVID-19 pandemic, including supply chain disruptions, increased demand, and volunteer shortages. To address these issues, effective solutions, including the management of food donations and volunteer scheduling, were proposed. Our findings have practical implications for decision-makers in food bank management, highlighting the importance of adopting evidence-based solutions. Finally, Limitations and prospective research directions in food bank management are discussed, with an emphasis on the need for ongoing research in this crucial area.

## Introduction

Food insecurity is a growing problem throughout the world, with nutritional shortages affecting more than one billion people. In 2015, the United Nations General Assembly set “zero hunger” as the second sustainable development goals (SDGs). Food insecurity occurs when people in need do not have enough food. According to the reports, these people face significant health and social issues such as a lack of safe and nutrient-rich foods and depression as a result of the social stigma associated with food insecurity [18, 59]. Eliminating hunger and achieving food security, also known as “Zero Hunger,” is a major global challenge [24]. Food insecurity affects at least 155 million people in 50 countries, and the COVID-19 pandemic has exacerbated the problem in many regions of the world [33].

The COVID-19 pandemic, as well as social distancing measures to slow its spread, have wreaked havoc on economies and food systems both globally and locally, with serious implications for food security [73]. Food shortages, a loss of disposable household income, higher food costs, and dietary changes are just a few consequences of the COVID-19 pandemic [95]. As a result of the global pandemic, more than 140 million people were predicted to be living in extreme poverty in 2020, a 20% increase from the previous year, and food insecurity increased dramatically [56]. Unfortunately, the COVID-19 pandemic is expected to exacerbate food insecurity, malnutrition, and obesity, potentially exacerbating health and social inequalities [45].

Despite the importance of food banks in addressing food insecurity, there is a lack of research on the application of OR methodologies in food bank operations. OR methodologies, such as optimization, and DP, have been used to study various aspects of supply chain management and logistics, including inventory management, transportation optimization, and warehouse design [4]. However, to our knowledge, no study has summarized the OR methodologies utilized by food bank operations. The application of OR methodologies in food bank operations could help optimize supply chain management, reduce operational costs, and increase efficiency, thereby improving the overall effectiveness of food banks in addressing food insecurity. Furthermore, there is a need for such research to better support food bank managers in adapting to the COVID-19 pandemic.

During the COVID-19 pandemic, governments implemented new policies and funding programs to aid food bank sectors in meeting the food security needs of the community. For instance, the United States Department of Agriculture (USDA) initiated a box program to assist the Los Angeles Regional Food Bank in maintaining its operations during the global pandemic [10]. With this additional funding and assistance, the food banks were able to establish themselves as a leading force in the ongoing pandemic’s fight against hunger.

Extant research on food bank operations and food insecurity have identified four main subject areas: food safety, user’s perception, food insecurity, and food bank operations. According to Table 1, there are several attends that offer review articles, surveys, and overviews within the food bank research. Food banks operations have been identified as one of the most intriguing aspects of food banks that have attracted the interest of researchers [4, 13, 68, 92]. Tarasuk et al. [92], for instance, conducted a survey to assess the factors that influence food bank operations in five Canadian cities. In another research, Booth and Whelan [13] investigated the operations and development of food banks in Australia using three analytical questions to assess social issues. McIntyre et al. [68] analyzed 33 articles on food bank operations published between 1998 and 2014 to promote policies that aid the operations of food banks in various counties. Finally, a study published by Ataseven et al. [4] focused on the human role in food bank operations by providing an intellectual framework to examine the human, managerial, and social capital background of food bank supply chain integration.

In response to this gap in the literature, the purpose of this paper is to conduct a comprehensive review of the existing research on the application of OR methodologies to food bank operations. Particularly, we aim to answer the following two important research questions:

- RQ1. How does extant literature address issues related to food bank operations in terms of OR methodologies?
- RQ2. How can the existing knowledge support food bank managers in adapting food banks amid the COVID-19 pandemic, and what are the potential future research opportunities? By addressing these research questions, this paper aims to contribute to the literature on food bank operations and OR methodologies, as well as to provide food bank managers with practical insights to help them adapt to the ongoing COVID-19 pandemic. Overall, this paper emphasizes the urgent need for additional research on the application of OR methodologies in food bank operations. By providing a comprehensive literature review and identifying potential future research opportunities, this paper aims to stimulate further research in this area and provide food bank managers with practical insights to help them address the challenges posed by the COVID-19 pandemic.

The remained parts of this article are structured as follows. Section “Review methodology” describes the review methodology of this systematic review. Section “Descriptive analysis of the review database” explicates the descriptive analysis of the review database. In Section “Applications of OR methods in food bank operations”, a comprehensive analysis of the researches used OR methods is provided. Section “Food bank operations under COVID-19 disruption” highlights the application of OR methods in mitigation of food bank operation disruption during COVID-19 pandemic. Following the concluding remarks and future directions in Section “Conclusion”.

**Table 1:** Review papers related to food bank operations

Reference	Article type	Subject area					Survey period
(sorted chronologically)		Food safety	Users’ perception	Food insecurity	Food bank operations	Solution techniques	
Makhunga et al. [65]	Review	$\checkmark$					2004–2018
Middleton et al. [70]	Review		$\checkmark$				1995–2015
Ataseven et al. [4]	Survey				$\checkmark$		1986–2014
Bazerghi et al. [9]	Review			$\checkmark$			1998–2015
McIntyre et al. [68]	Review				$\checkmark$		1998–2014
Loopstra and Tarasuk [60]	Survey			$\checkmark$			1990–2014
Booth and Whelan [13]	Overview				$\checkmark$		1980–2014
Tarasuk et al. [93]	Survey			$\checkmark$			1986–2012
Tarasuk et al. [92]	Survey				$\checkmark$		1986–2014
Blessley and Mudambi [11]	Review			$\checkmark$			2018–2020
Simmet [87]	Survey			$\checkmark$	$\checkmark$		2020–2020
Rivera et al. [84]	Review			$\checkmark$	$\checkmark$		2000–2022
Our study	Review				$\checkmark$	$\checkmark$	2000–2023

## Review methodology

**Fig. 1:**
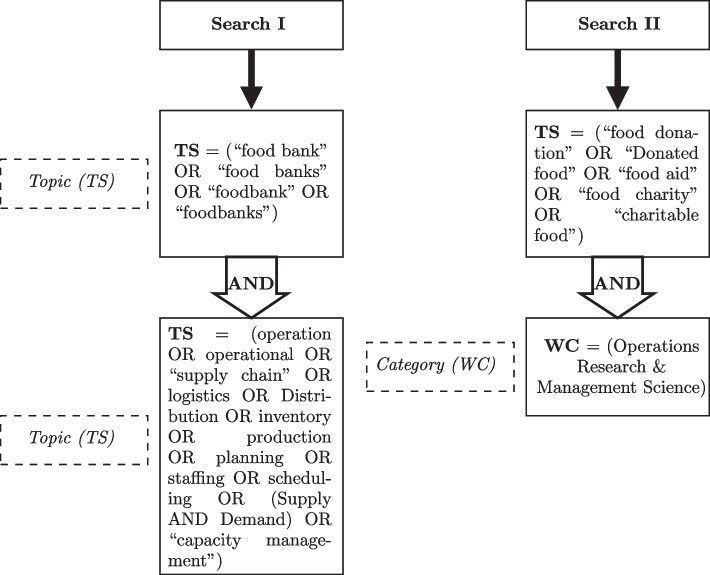
Searching strategy

**Fig. 2:**
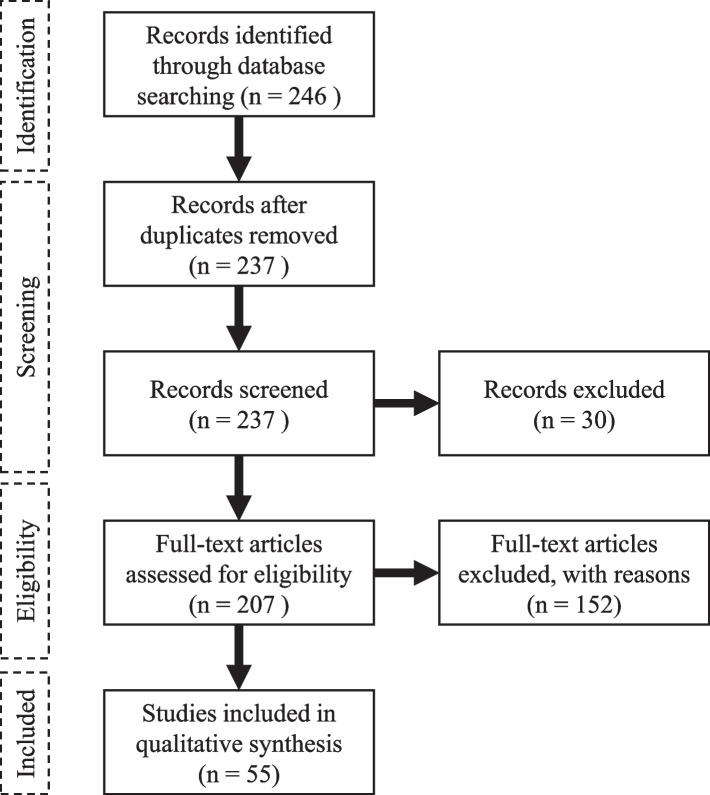
PRISMA flow chart

To conduct an extensive assessment and gather relevant papers, we followed the Preferred Reporting Items for Systematic Reviews and Meta-Analyses (PRISMA) approach [71]. The PRISMA method methodology comprises four-stages: “Identification”, “Screening”, “Eligibility” and “Included.” The searches were first carried out in January 2021 and updated in April 2023 using the Web of Science (WoS) database. Specifically, literature published from 2000 to 2023 were evaluated. Figure 1 depicts the process of choosing and screening literature via the PRISMA method to arrive at a final selection of 55 articles for a comprehensive study. Figure 1 provides a visual representation of our search process and the number of articles identified at each stage.We employed two search methods to identify relevant articles. In the first search (Search I), we used keywords such as “food bank,” “operations management,” “supply chain,” “distribution,” and “production” to identify articles related to our research topic. Our initial search resulted in 217 hits. In the second search (Search II), we searched for articles in the Web of Science’s OR &MS category that contained food bank-related keywords, such as food charity, food donation, and food aid. The database searches resulted in 29 hits. According to Fig. 2, after removing duplicates and screening for relevance (excluded non-English and irrelevant articles), we included 55 articles in our comprehensive study. Our final selection of articles underwent a thorough analysis to ensure that they met the included criteria and were relevant to our research question. To ensure the accuracy and integrity of the final selection, the elimination of duplicate records was performed manually. Each record was meticulously examined to identify and eliminate any duplicate entries, ensuring that each article was only counted once in the analysis. In order to determine the relevance of the articles, specific inclusion and exclusion criteria were implemented during the screening phase. Articles written in languages other than English were excluded from the study to maintain language uniformity and facilitate effective comprehension of the results. In addition, the article’s content was meticulously evaluated to determine its direct relevance to our research query. Articles that did not directly relate to the topic under investigation or did not offer substantive insights were deemed irrelevant and subsequently eliminated from the final selection.

**Fig. 3:**
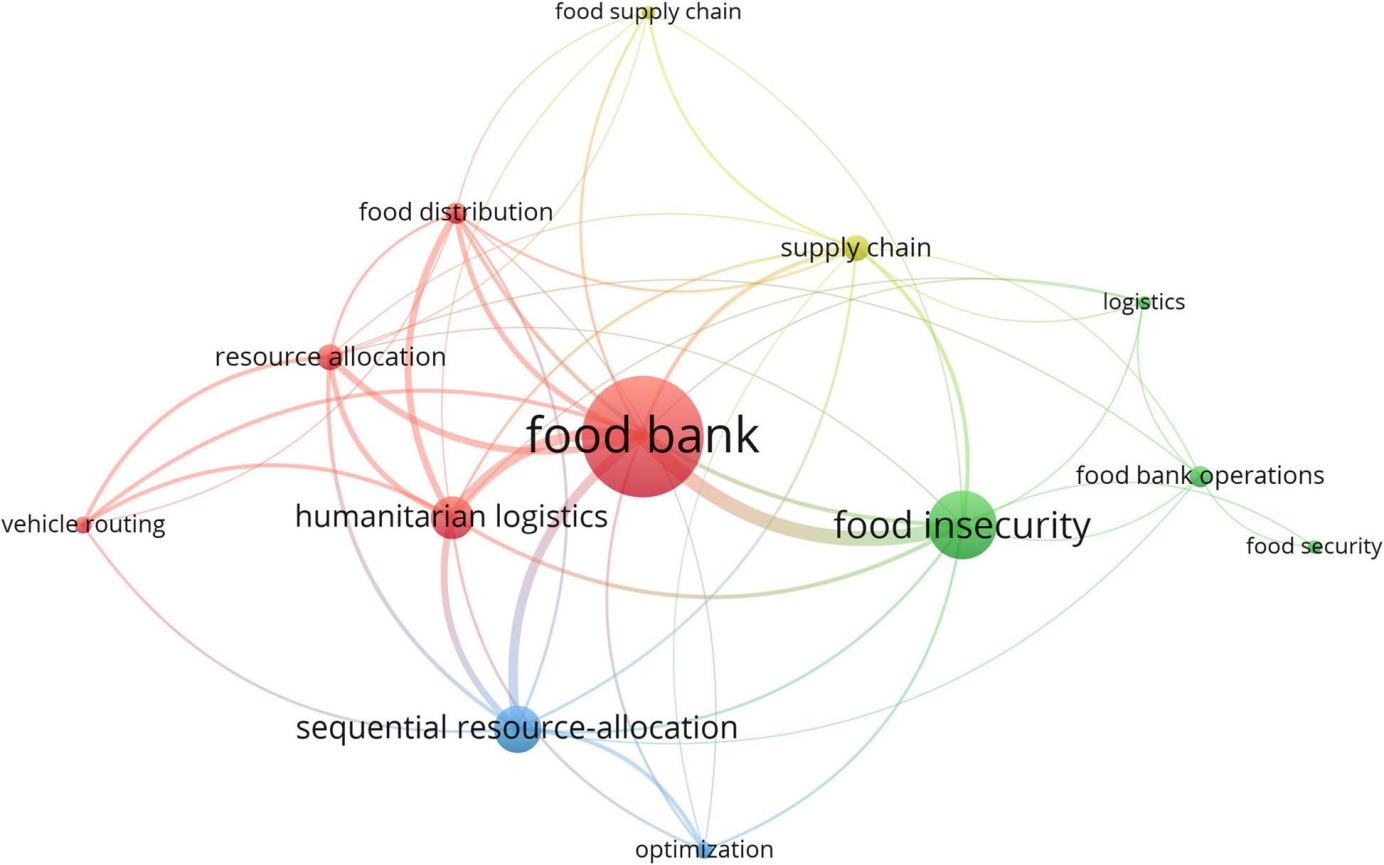
Results from Bibliometric analysis

The inclusion of 55 articles in our comprehensive study represents the culmination of a meticulous and systematic process. In order to identify the recurring terms of these articles’ “Title” and “Abstract” and to construct a term co-occurrence map using Network analysis and graphical investigation, bibliometric analysis, similar to that of Corallo et al. [21] was conducted. Minimum frequency of selected recurring terms is six. Therefore, according to the comprehensive counting method, 323 terms recurred, whereas only 13 terms recurred within the last six occurrences. Each of the thirteen keywords in the network was represented by a distinct color. Using a VOSviewer, Fig. 3 illustrates the numerous clusters, the evolution of the general terms network over time, and the emphasis on the food bank node. While there are numerous articles on “food banks” and “food insecurity,” there are insufficient articles on operations such as food distribution, vehicle routing, resource allocation, etc., as indicated by Fig. 3, While there are numerous articles on “food banks” and “food insecurity’, there are insufficient articles on operations such as food distribution, vehicle routing, resource allocation, etc. This demonstrates the necessity of including food bank operations in a future study.

## Descriptive analysis of the review database

**Fig. 4:**
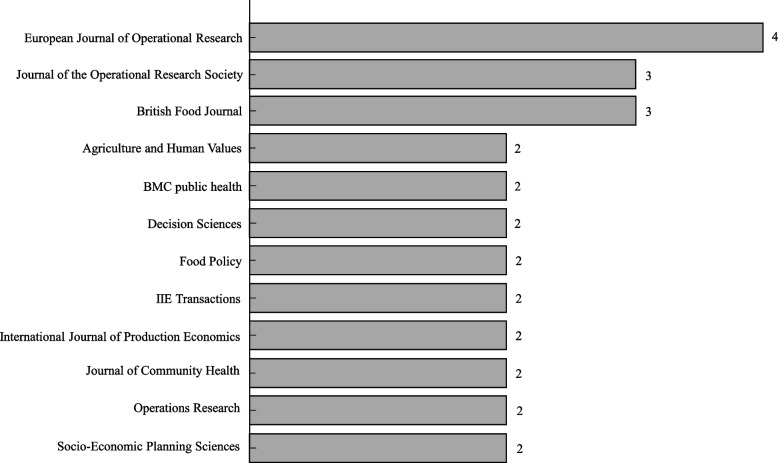
Distribution of articles by contributing journals

To conduct a descriptive analysis of the review database, we tallied the number of articles published by contributing journals, the level of spatial differentiation, and the methods employed in the articles. The present study analyzed 55 articles that are contributed from a variety of 38 journals (45 Q1, 8 Q2, 1 Q3, and 1 Q4 papers according to Scimago Journal Country Rank (SJR) as shown in the Table 2). However, Fig. 4 displays the journals with multiple articles, which account for 51% (28 articles) of the 55 papers published in the 12 contributing journals. Seven out of twelve journals of Fig. 4 are classified as OR &MS category, namely *European Journal of Operational Research*, *Decision Science*, *IIE Transactions*, *International Journal of Production Economics*, *Journal of the Operational Research Society*, *Operations Research*, and *Socio-Economic Planning Sciences*. The *European Journal of Operational Research* contributed the most articles with four, followed by *Journal of the Operational Research Society* with three articles, and the other OR &MS journals with two articles each.

**Table 2:** Collected papers information according to SJR

Reference	Publisher	ISSN	Impact Factor	JCI Quartile
Martins et al. [67]	ELSEVIER	0925-5273	10.54	Q1
Schneider and Nurre [86]	Elsevier BV	03050483	2.74	Q1
Orgut et al. [78]	Elsevier	03772217	2.35	Q1
Buisman et al. [16]	Elsevier	03772217	2.35	Q1
Fianu and Davis [31]	Elsevier	03772217	2.35	Q1
Hindle and Vidgen [42]	Elsevier	03772217	2.35	Q1
Reihaneh and Ghoniem [83]	Taylor and Francis Ltd.	01605682	0.88	Q1
Ghoniem et al. [37]	Taylor and Francis Ltd.	01605682	0.88	Q1
Blackmon et al. [10]	Wiley-Blackwell	10591478	3.34	Q1
Balcik et al. [7]	Taylor and Francis Ltd.	24725854	1.14	Q1
Orgut et al. [76]	Taylor and Francis Ltd.	24725854	1.14	Q1
Orgut et al. [77]	Taylor and Francis Ltd.	24725854	1.14	Q1
Loopstra et al. [61]	BioMed Central Ltd.	14712458	1.16	Q1
Tarasuk et al. [92]	BioMed Central Ltd.	14712458	1.16	Q1
Ataseven et al. [4]	Wiley-Blackwell Publishing Ltd	00117315	1.67	Q1
Sawaya III et al. [85]	Wiley-Blackwell Publishing Ltd	00117315	1.67	Q1
Bryan et al. [15]	Springer Netherlands	00945145	0.89	Q1
Rancourt et al. [82]	Elsevier Ltd.	03050548	1.86	Q1
Wills [96]	Elsevier BV	03069192	1.93	Q1
Lee et al. [58]	Elsevier BV	03069192	1.93	Q1
Bazerghi et al. [9]	Springer Netherlands	00945145	0.89	Q1
Sonmez et al. [90]	John Wiley and Sons Inc.	00029092	1.86	Q1
Brock and Davis [14]	Elsevier Ltd.	09574174	2.07	Q1
Davis et al. [22]	Elsevier Ltd.	00380121	1.1	Q1
Strong [91]	Elsevier BV	00167185	1.42	Q1
Makhunga et al. [65]	BioMed Central Ltd.	20464053	0.95	Q1
Eisenhandler and Tzur [29]	INFORMS Institute	00411655	2.81	Q1
Lindberg et al. [59]	Wiley-Blackwell Publishing Ltd	09660410	0.82	Q1
Solak et al. [89]	Springer Netherlands	02545330	1.17	Q1
Nair et al. [72]	Elsevier Ltd.	00380121	1.10	Q1
Marthak et al. [66]	Springer Netherlands	0921030X	0.7	Q1
Loopstra and Tarasuk [60]	Cambridge University Press	14747464	0.53	Q1
McIntyre et al. [68]	Springer Netherlands	0889048X	1.01	Q1
Bacon and Baker [6]	Springer Netherlands	0889048X	1.01	Q1
Gonzalez-Torre et al. [39]	Springer New York	09578765	0.84	Q1
Rancourt et al. [82]	Elsevier Ltd.	03050548	1.86	Q1
Blessley and Mudambi [11]	Elsevier Ltd.	00198501	2.21	Q1
Middleton et al. [70]	Academic Press Inc.	01956663	0.99	Q1
Davis et al. [23]	ELSEVIER	0925-5273	10.54	Q1
Ataseven et al. [4]	INFORMS Institute	0030364X	3.62	Q1
Hasnain et al. [40]	Elsevier Ltd.	22124209	1.10	Q1
Kaviyani-Charati et al. [53]	Elsevier Ltd.	03608352	1.78	Q1
Firouz et al. [32]	Taylor and Francis Ltd.	01605682	0.88	Q1
Eisenhandler and Tzur [28]	INFORMS Institute	0030364X	3.62	Q1
Ataseven et al. [5]	Emerald Group Publishing Ltd.	20426747	3.02	Q1
Ortuno and Padilla [79]	Omnia Publisher SL	20130953	0.44	Q2
Rivera et al. [84]	Emerald Group Publishing Ltd.	20426747	0.73	Q2
Ogazón et al. [74]	MDPI AG	22277390	0.54	Q2
Simmet [87]	Taylor and Francis Ltd.	19320248	0.46	Q2
Ahire and Pekgün [1]	INFORMS Institute	1526551X	0.66	Q2
Booth and Whelan [13]	Emerald Group Publishing Ltd.	0007070X	0.61	Q2
Tarasuk et al. [93]	Emerald Group Publishing Ltd.	0007070X	0.61	Q2
Thompson et al. [94]	Thomas A. Lyson Center	2152-0801	2.2	Q2
Gomez-Pantoja et al. [38]	Springer Netherlands	1134-5764	1.85	Q3
Douglas et al. [26]	Aimsm Press	2327-8994	1.8	Q4

**Fig. 5:**
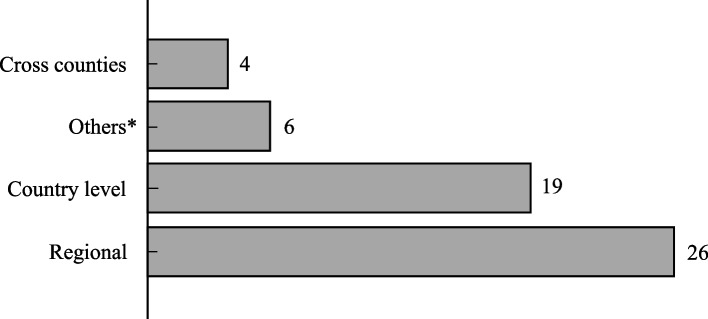
Distribution of articles by the level of spatial differentiation. (Others category includes the research conducted by Booth and Whelan [13], Bazerghi et al. [9], McIntyre et al. [68], Reihaneh and Ghoniem [65, 83], and Middleton et al. [70] where the first five papers are review papers and the data for the last study is randomly generated.)

Figure 5 illustrates the degree of spatial differences in three levels: regional, country-level, and cross country. The evaluations in 26 articles are in the regional level, which means they are based on average data from particular geographic areas. The evaluations in 19 articles are in the country level, which means they are based on average data from multiple geographic areas of a country. Finally, the evaluations in four articles are in the cross-country level, which means they are based on average data from multiple countries.

**Fig. 6:**
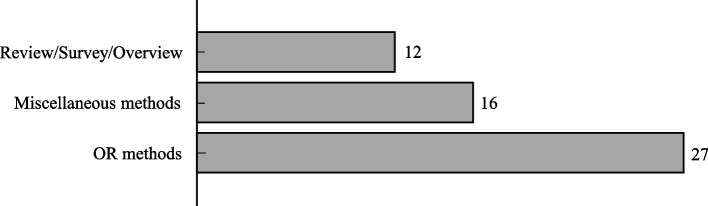
Distribution of articles by the methods

According to the aim and context of the evaluation, the methods used in food bank problems can be classified into three broad categories: review/survey/overview, miscellaneous, and the OR methods (Fig. 6). There are also 12 review/survey/overview articles in the literature as listed in Table 1. There are also 16 articles using miscellaneous methods such as Monte-Carlo simulation [5], neural network [14], Geographic Information System (GIS) [6], time series [85], and statistical and empirical methods [15, 23, 26, 35, 40, 42, 59, 61, 72, 91, 94, 96]. In this group, 12 articles [5, 9, 11, 13, 23, 60, 65, 69, 72, 84, 87, 92, 93] were published in OR &MS journals. Finally, the OR methods are the largest category and are employed in 27 out of 55 articles. The OR methods, consist of Linear Programming (LP), Integer Programming (IP), Dynamic Programming (DP), and Data Envelopment Analysis (DEA). We will discuss the applications of OR methods in food bank operations in detail in the next section.

## Applications of OR methods in food bank operations

This section examines the application of OR methods in food bank operations. Table 3 illustrates a detailed paper-by-paper analysis of applied OR methods as well as objectives and the sources of uncertainty of each study. In our analysis, we found that there are four OR methods used in food bank operation research: LP [58, 75, 77, 78], IP [1, 10, 16, 28, 29, 37, 38, 66, 67, 79, 82, 83, 86, 89, 90], DP [3, 7, 31], and DEA [39]. In the remainder of this section, we provide more detail assessment in OR methods. Section “Problem type” investigates the application of OR methods to a variety of food bank operation problems. In Section “Operation research objectives”, the objective of food bank operations is discussed. Finally, Section “Foodbank operations under uncertainty” investigates the sources of uncertainty and the techniques used to address these uncertainties.

**Table 3:** OR methods in food bank operations

Reference	Method	Problem Type				Single-objective/Multi-objective	Deterministic/Uncertain
		Distribution	Inventory	Facility planning	Scheduling		
Marthak et al. [66]	MILP	$\checkmark$				Multiple	Uncertain
Blackmon et al. [10]	MILP	$\checkmark$				Single	Deterministic
Gomez-Pantoja et al. [38]	MILP	$\checkmark$				Single	Deterministic
Martins et al. [67]	MILP		$\checkmark$	$\checkmark$		Multiple	Deterministic
Schneider and Nurre [86]	MILP				$\checkmark$	Multiple	Deterministic
Ogazón et al. [74]	MILP	$\checkmark$				Multiple	Deterministic
Kaviyani-Charati et al. [53]	MILP	$\checkmark$	$\checkmark$	$\checkmark$		Multiple	Uncertain
Eisenhandler and Tzur [28]	MILP	$\checkmark$				Single	Deterministic
Eisenhandler and Tzur [29]	MILP	$\checkmark$				Single	Deterministic
Buisman et al. [16]	MILP		$\checkmark$			Single	Uncertain
Ata et al. [3]	DP				$\checkmark$	Single	Uncertain
Orgut et al. [78]	LP	$\checkmark$	$\checkmark$	$\checkmark$		Multiple	Uncertain
Ahire and Pekgün [1]	MILP		$\checkmark$			Single	Uncertain
Reihaneh and Ghoniem [83]	MILP	$\checkmark$				Single	Deterministic
Fianu and Davis [31]	DP	$\checkmark$				Single	Uncertain
Lee et al. [58]	LP				$\checkmark$	Single	Uncertain
Ortuno and Padilla [79]	MILP	$\checkmark$				Single	Uncertain
Orgut et al. [77]	LP	$\checkmark$				Single	Uncertain
Gonzalez-Torre et al. [39]	DEA				$\checkmark$	Single	Deterministic
Rancourt et al. [82]	MILP	$\checkmark$				Single	Deterministic
Sonmez et al. [90]	MILP				$\checkmark$	Single	Uncertain
Orgut et al. [75]	LP	$\checkmark$		$\checkmark$		Single	Deterministic
Firouz et al. [32]	LP	$\checkmark$				Single	Uncertain
Balcik et al. [7]	DP	$\checkmark$				Multiple	Uncertain
Ghoniem et al. [37]	MILP		$\checkmark$			Single	Deterministic
Solak et al. [89]	MILP	$\checkmark$				Single	Deterministic

### Problem type

According to our analysis, food bank operation problems exist in four areas: distribution management, inventory management, facility planning, and scheduling. The remainder of the section discusses the OR methods used in each of these problems.

#### Distribution management.

Food banks deal with various operational problems in a daily basis. Distribution management problems are the most common challenges in food bank operations that attain many attention in the literature. Table 3 shows that there are 17 articles that examine food bank distribution problems. The objective of food bank distribution management is to collect, sort, and distribute food donations in order to connect individuals and businesses with excess food to those in need [64]. The expression “distribution management” was first used by Eilon, Watson-Gandy and Christofides in 1971 [57]. Since then, it has been extensively studied in the OR literature. We next review OR methods to management distribution problems in food banks.

Most of the distribution studies benefit from MILP methods. The MILP methods belong to the mathematical optimization problem with some or all integer variables. Eisenhandler and Tzur [29] proposed an MILP optimization model to facilitate the pickup and distribution decisions via equitable allocations to the different food bank agencies. Similarly, [89] extended an MILP to make the decisions of selecting the delivery location as well as assigning food bank agencies and the required vehicles to that location. Rancourt et al. [82] investigated food distribution in Kenya via solving facility location problem as an MILP formulation in order to identify a set of distribution centers. Gomez-Pantoja et al. [38] developed an MILP formulation to optimize their decisions including inventory, purchases, product-beneficiary, and balanced nutrition. In another research, [83] proposed a multi-start optimization-based heuristic to cope with the food bank distribution problem and to determine vehicle routing and demand allocation decisions. Blackmon et al. [10] developed an MILP embedded in Decision Support Systems (DSS) to decide their inventory management policies during the COVID-19 pandemic. Finally, [66] proposed a two-stage stochastic MILP programming model to determine the location of food banks’ supplies.

There are few articles in the literature that employed LP models to address food bank distribution management problems. For example, [77] proposed a stochastic LP model for food bank distribution with an uncertain capacity. The DP is the another method used by [7] and [31] to ensure equitable distribution of food bank supplies [7, 31].

#### Facility planning.

Another type of food bank problem is facility planning. Facility planning assists food bank operators to meet organisational requirements, orders, and deadlines. In general, food banks coordinate vehicles to collect local donations and deliver food to rural partner agencies. It is crucial for these vehicles to ensure food are stored in a safe environment during the collection and delivery processes. Table 3 shows three articles evaluating the facility planning problem of food banks. Orgut et al. [75] proposed a LP model for food bank distribution and inventory management that is equitable and effective. In another study, [78] extended the model of [75] by considering uncertain capacity using robust optimization methods to deal with distribution, inventory, and facility planning problems of food banks. Martins et al. [67] also attempted to propose a MILP model to deal with inventory management and facility planning in a more sustainable, economical, and environmental way.

#### Volunteers scheduling.

Volunteers make up the majority of the personnel at food banks. Enrolling, scheduling, and managing volunteers are critical as demand increase, causing challenges in food bank sectors. In this respect, almost 21% of the articles (5 out of 24 articles) in Table 3 are concerned with the scheduling challenges faced by food banks.

According to our analysis, OR methods applied to cope with food banks scheduling studies are MILP, LP, DP, and DEA. In a couple of food banks studies [86, 90], researchers presented benefits from MILP models on volunteering scheduling. Sonmez et al. [90], for instance, introduced a stochastic MILP model to determine and schedule the gleaning operations of a food bank. Similarly, [58] extended a stochastic model to identify the operating efficiencies of the gleaning operations. However, they have applied an LP optimization model to determine the appropriate scheduling of food banks. The schedule efficiency of the partner agency audit is also enhanced via a multi-criteria MILP model for food banks is developed by [86].

Aside from the MILP and LP models described above, food bank researchers used other methods to deal with scheduling problems including DP and DEA [3, 39]. DEA-based efficiency analysis is also used by [39] to determine the effective and ineffective food banks based on their operations. Ata et al. [3] proposed a model to characterize the gleaning operations in which the uncertainties are considered as dynamic variables.

#### Inventory management.

Food banks are supported by a variety of supply sources that vary in terms of quantity and delivery time. Therefore, donation variation and demand changes caused inventory fluctuations in some food bank sectors. Hence, applying a proper inventory management policy is necessary in food bank operations. There are limited studies in the literature that examined inventory management of food banks and they are integrated by other food bank problems. Ghoniem et al. [37] provided a MILP model to address the food bank’s vehicle routing problems and demand allocation. Davis et al. [22] proposed a MILP model with the objective of minimising the number of food delivery points and total distance travelled throughout the planning horizon. Ahire and Pekgün [1] suggested an optimization framework to assist Harvest Hope food bank in Carolina. Buisman et al. [16] have also used the MILP approach to handle donation management and menu planning in order to determine which part of food bank donations should be accepted.

### Operation research objectives

The micro-analysis of different studies leads us to classify the collected papers into two classes in terms of the objective numbers: single and multiple objective functions. One of the major objectives of food bank studies is cost reduction. For example, [89] proposed a model to decrease food bank operation-related costs such as vehicle routing, agency travel, fixed-site selection, and agency travel. Ghoniem et al. [37] helped to assess the weighted average distance travelled by food bank vehicles and clients. Rancourt et al. [82] attempted to reduce the overall welfare cost of all regional food distribution stakeholders. Reihaneh and Ghoniem [83] intended to reduce customer travel costs. Buisman et al. [16] also attempted to minimize the cost that went into purchasing ingredients.

Achieving the maximum gleaned value is another objective considered by the food bank researchers. The objective of [90]’s research was to estimate how many gleaning trips would be required to solve the problem facing the Southern Tier Food Bank in New York City. Ortuno and Padilla [79] tried to maximize the quantity of energy content amount of food in the food banks. Lee et al. [58] proposed a model for evaluating gleaning producers’ operational performance by maximising the expected total gleaned value. Besides, [3] proposed a dynamic model to maximize the average net payoff of the food gleaned amount.

There are some researches exploring qualitative objective in their studies such as equal services, equal distribution, equity policy, and social awareness. For instance, [7] addressed a vehicle-allocation problem in order to provide an equal service and to decrease food bank sector waste. Orgut et al. [75] sought to reduce total undistributed food bank supplies while ensuring a perfectly equal distribution. Ahire and Pekgün [1] utilized a model to calculate the maximum number of promotional events such as media events to raise food and dollar donations of food banks. Fianu and Davis [31] seek to maximize the equity by minimizing equity policy. Similarly, [29] examined ways to maximize the total amount distributed by the food bank vehicles. Eisenhandler and Tzur [28] also formulated the logistic problem of food banks to maximize the amount of food delivery in the distribution network. Gomez-Pantoja et al. [38] introduced a model for the food bank to maximize the score coming from nutrients of the food. Finally, [10] made an attempt to ease food bank shortfalls of various foods of food banks.

While most food bank researches focused on only one objective, some researchers focused on multiple objectives [22, 66, 67, 78, 86]. Costs, distance, food amounts, audit time, and food spoilage risk are some objective examples explored by the researchers. Davis et al. [22] proposed a model that reduce the number of food delivery points while minimising total travel distance over the planning horizon. Orgut et al. [78] provided two models to maximise food bank deliveries while reducing food deterioration. Martins et al. [67] suggested a model to reduce overall cost, food waste, and maximise equitable distribution of donated food. Schneider and Nurre [86] also proposed a model in which the overall distance travelled is minimized, route numbers are minimized, and audit time is maximized. Finally, demand coverage, weighted distance between demand and supply locations, and distribution distance were all considered by the model proposed by [66].

### Foodbank operations under uncertainty

In order to address the issue of uncertainty and incomplete data, different OR techniques have been applied by the food bank researchers (Table 4). Stochastic optimization (SO) is the most common technique to address uncertainty among available techniques. Such technique assumed that either distribution or a set of possible scenarios of uncertain data are known. Also, [58] and [3] showed that one can study food bank operations in dynamic setting. There are two main source of uncertainties in food bank operations: donated supplies and labour capacity.

**Table 4:** Foodbank operations under uncertainty

Reference	Technique	Uncertainty set	Static/Dynamic	Source of Uncertainty
Marthak et al. [66]	SO$^\dag$	Scenario	Static	Supplies, demand, and food
Ata et al. [3]	SO	Distribution	Dynamic	Food and labor
Buisman et al. [16]	SO	Scenario	Static	Shelf life and labor
Orgut et al. [78]	RO$^\ddag$	Distribution	Static	Food and capacity
Fianu and Davis [31]	SO	Distribution	Static	Supply
Lee et al. [58]	SO	Scenario	Dynamic	Food and labor supply
Ortuno and Padilla [79]	FO$^*$	Fuzzy	Static	Energy, and food volume and weight
Orgut et al. [77]	SO	Distribution	Static	Food and agencies capacity
Sonmez et al. [90]	SO	Scenario	Static	Food and labor
Firouz et al. [32]	SO	Scenario	Static	Capacities of coutries to receive and handle food
Kaviyani-Charati et al. [53]	SO	Scenario	Static	Demand for each food type

$^\dag$ SO: Stochastic Optimization, $^\ddag$ RO: Robust Optimization, $^*$: FO: Fuzzy Optimization

## Food bank operations under COVID-19 disruption

During the COVID-19 pandemic, food banks faced several operational challenges due to a dramatic increase in demand and a considerable loss in volunteers [69]. One of the most serious issues caused by COVID-19 is the disruption in food supply and distribution. An US study showed that among 200 American food banks, 98% experienced demand increase and 59% face inventory reduction through emerging COVID-19 pandemic [44]. As a consequence, food banks had to modify their regular supply and distribution routine to meet the new safety measures and the increasing demand [54]. Another problem that food banks faced during the COVID pandemic was a lack of staff and volunteers. As food bank operations were highly dependent on volunteers and the majority of food bank volunteers were older people over the age of 70, the new public health concerns often kept these individuals away from volunteering at the food banks [81].

### Challenges

Since the World Health Organization announced the COVID-19 pandemic in March 2020, food banks around the world deal with the disproportionate impacts of the global epidemic [10, 17, 100]. The temporal job loss and closure of businesses turned a number of households to food banks. Food insecurity also leads to other significant social and health problems such as depression and malnutrition [62]. In addition to addressing challenges during the pandemic, many food bank operators started worrying about the post-pandemic period as they have no idea how long they could meet these ever-growing food demands [43]. The following sections present some of the identified food bank challenges during and after the COVID-19 pandemic.

#### Lack of Supply.

The COVID-19 pandemic has proven to be one of the most disruptive global events that has resulted in severe food insecurity. The World Bank recorded an increase of 110 million people deemed food insecure due to the pandemic [27]. The resulting food disruptions across the global and local supply chain have caused supply decrease and food demand increase. In addition to the reductions in agricultural production and supply chain disruptions, the panic buying behavior exhibited by consumers during the pandemic has also contributed to a significant rise in demand and a reduction in the food supply. These disruptions have challenged the principal purpose of food banks, and the food bank sectors were faced with extreme pressure due to the pandemic. Therefore, food bank sectors changed their operations to cope with the difficulties of the COVID-19 pandemic. The mentioned operational changes have added extra financial burdens to food banks and called for the food bank sectors to implement a recovery plan, especially for the post-pandemic era of COVID-19.

#### Staff and volunteer shortage.

Another pandemic-induced challenge we identified is related to the food banks’ staff and volunteering. Food banks staff consist of volunteer and paid workers. Volunteers at food banks are mostly elderly people over the age of 70 who are willing to assist those living in poverty [30]. As a result of the COVID-19 epidemic, volunteers were turned away from food banks due to public health and public safety regulations regarding social distance and food service [81]. In this sense, food banks were unable to adequately sustain its operations due to a significant reduction in personnel. The increased demands during the pandemic also required additional personnel therefore further challenge food banks operation. Some recent study showed that several food banks were forced to close temporarily or permanently due to the lack of human resources [81]. It is therefore essential to carefully examine these challenges, and provide some practical recommendations to overcome these significant difficulties.

#### PPE supplies and new safety protocol.

In addition to maintaining the essential services to those in need, the local food banks were concerned about the health and safety of the frontline personnel during the COVID-19 pandemic [43]. Many food banks modified their operations process to meet with the new safety protocol and public health concerns. For instance, food banks now are required to provide their staff with personal protective equipment (PPE) such as masks, gloves, shields, and hand sanitizer.

While PPE minimized the chance of the COVID virus spreading among staffs who were working at the food banks, wearing a PPE kit for a long period of time caused certain level of discomfort such as excessive sweating, which may result in headaches and breathing difficulties. Also, PPE has an encapsulating and impermeable characteristic that inhibits heat loss, which increases heat stress when applied to the extra weight and restrictions in movement [19]. Hence, it is essential to provide appropriate solution such as regular breaks and additional rest areas for food banks staff and volunteers to minimize these impacts.

### Supply chain response to COVID-19 disruption

The food bank supply chain, like other supply chain systems, has been disrupted by COVID-19 significantly. Such disruption reveals the need of having a proper approach to food bank supply chain design, one that is effective in normal conditions but stronger in a crisis. Based on a recent report from Mckinsey and Company, there are three phases to make the supply chains more resilient during disruptive events such as the COVID-19 pandemic [63]. In their review of extant OR studies on supply chain disruption and dynamics during the COVID-19 pandemic, [48] recommended several strategies for dealing with this disruption before, during, and after the epidemic. Each key phases in preparing for the potential COVID-19 disruption will be described in details. Remark that the supply chain disruption methods available in the literature can also be used for food bank supply chain.

#### Rapid response.

Nowadays, the emergence of the COVID-19 epidemic played havoc on food supply chains. COVID-19 pandemic caused a massive disruption which is the initial long-lasting supply chain crisis for the recent decades [49]. Responding to this pandemic has been a difficult challenge, especially for food banks, as they should have an adaptive behavior to deal with both demand and supply sides shocks. Besides, disruption in demand and supply can lead to other problems such as inventory and safety problems. The risk of supply chain disruption is increased significantly when the demands, supply, or both are vulnerable. Hence, implementing an effective management system to respond to the supply chain disruption quickly is crucial in food bank sectors as they should quickly adapt to the changes caused by the disruptions. In this regard, many researchers attempted to examine the reactions of supply chains during the pandemic [8, 10, 25, 47, 99]. Ivanov [47] and Yang et al. [99] have investigated supply chain adaptive behaviors during the pandemic. The initial responses of main farming and food systems in 25 Asian nations to COVID-19 are also assessed by [25]. Besides, [12] proposed a study to identify and prioritize the critical steps of distribution response to mitigate the disruption impacts. Blackmon et al. [10] also developed a decision support system (DSS) framework to manage inventories during the COVID-19 pandemic.

#### Recovery plan.

Once managers have stabilized the rapidly shifting demand and supply flows to respond effectively to an emergency, they must devise plans to restore public trust and confidence. According to [8], supply chain disruption management entails the forecasting of risks and the deployment of methods to mitigate the interruption. By implementing such recovery methods, the firm can respond rapidly to supply chain interruptions and be prepared for future disasters. These recovery strategies can be divided into proactive and reactive strategies to tackle any form of supply chain disruption. In a research conducted by [98], reactive and proactive methods were proposed for developing food sector resilience in response to the disruption caused by the COVID-19 pandemic. Both methods involve a robust implementation of risk management. Paul et al. [80] developed a reactive approach to recover from the supply distribution in a three-tier supply chain system. Besides, the resilience of operations is another critical element providing flexibility in recovery. Therefore, [41] identified the critical factors related to the resilience of 26 food system businesses and organizations in Baltimore. Similarly, [2] investigated the significant elements of a resilient food system that can deal with external shocks such as the COVID-19 epidemic. Kumar and Singh [55] also proposed a strategic framework for improving agricultural food supply chain resilience. Using a robust version of a mathematical programming model to assess the supply chain’s resilience and deal with disruptions is a perfect example of the OR method. For example, [36] suggested a resilient MILP location-allocation-inventory model for disrupted food supply chains, and they interpreted three resiliency strategies to the presented mathematical model. Xia et al. [97] also utilized a model based on a disruption recovery method employing a recovery time window with the intention of reducing costs.

#### Reconfiguration plan.

The COVID-19 pandemic showcased the vulnerability of the modern agricultural and food markets [25]. Food banks, as an essential unit for providing food and nutrition to the vulnerable and marginalized population, need to critically maintain a robust and adaptable food supply during the critical situation. Similar to other supply chains, food banks should use reconfiguration methods to make their supply chain operations more robust in the case of extreme catastrophes. These methods can help food bank sectors sustain the food bank sector’s operations as they transition back to pre-pandemic ways.

Although extant academic literature in supply chain management discusses supply chain disruptions mitigation strategies [e.g., 20, 34], the current pandemic outbreak has introduced an altogether different set of challenges that the existing literature does not provide a complete and concrete set of solutions. Supply chain disruption has been restudied since the emergence of COVID-19. For instance, [46] investigated at production-ordering behavior in a supply chain with interruption risks during recovery and post-disruption phases, as well as the impact of severe disruptions on production and distribution network design. Ivanov [49] presented a new concept called a viable supply chain (VSC), which can help businesses recover and rebuild supply networks following long-term disasters like the COVID-19 epidemic. In another research, [50] also suggested a framework for post-pandemic SC management, which included five strategic and operational aspects. Finally, simulation model proposed by [51] to assist managers in selecting the appropriate post-pandemic strategies for a supply chain. For further detail we refer to [52] and [52]. Singh et al. [88] discussed a development of resilient and responsive food supply chains to meet changing demand, as well as decision-making support for rerouting vehicles in areas with travel limitations. Kaiser et al. [88] examined the main components of reconstructing food systems in the post-pandemic era.

## Conclusion

In this study, we examined how OR methods have aided food banks. The analysis of the selected articles revealed various methods from OR were applied to address food bank operation challenges. Based on a comprehensive analysis of selected articles, it is evident that OR methodologies, such as IP, LP, DP, and DEA, have played a significant role in expanding the evaluation and providing effective solutions for food bank operations. The main conclusion of this study is that OR methods have proven to be invaluable tools in aiding food banks by offering practical and actionable solutions to their operational challenges. The utilization of optimization methods, which constituted the majority of the collected OR papers, demonstrates their effectiveness in enhancing food bank operations. The distinctive nature of these methods, focused on providing concrete solutions rather than mere potential solutions, further strengthens their relevance in addressing food bank challenges. Moreover, this study acknowledges the unique challenges posed by the COVID-19 pandemic and highlights the need to resolve these challenges effectively. By examining the food bank operation challenges before, during, and after the pandemic, the study provides insights into the effective methods and strategies that can be employed in such extraordinary circumstances. This in-depth analysis paves the way for implementing these methods to address pressing concerns that question the fundamental purpose of food banks.

This study is not without its limitations. One limitation of this research relates to the sample extraction process, which initially focused on journal articles, resulting in a small number of relevant studies being included. The inability to access databases such as PubMed and Scopus further constrained the sample extraction process, thereby restricting the study’s scope. To address this limitation and assure a more comprehensive analysis, future research should consider incorporating additional resources, such as conference proceedings and the aforementioned databases, to increase the number of relevant studies and enhance the scope of the analysis. The search term is an additional limitation of the present study. Due to the search term limitation, we may have overlooked studies employing other terms, even though we have considered some of the food bank-related key terms. Studies in the future should consider employing broader search terms to ensure a more comprehensive analysis of relevant studies. A further limitation is the review process. While we have considered non-English and duplicate papers to minimize potential biases, there may still be publication biases, as these are the only exclusion criteria considered during the selection process, and it may affect the study’s findings. Future studies should consider implementing PRISMA enhancements to reduce potential review biases. The last limitation relates to the scope of the study. This study is primarily concerned with the application of OR methodologies to food bank operational challenges. However, food banks confront a variety of other challenges not addressed in the manuscript, including addressing food insecurity at the community level, fundraising and donor management, and reducing food waste. In the future, studies should consider a deeper examination of food bank operations, including a broader range of challenges and potential solutions.

While this research sought to examine food bank challenges in the context of COVID-19 pandemic, further examination of food bank operations is required. For example, in addition to promised and continuous food supply, the inventory challenges can be addressed by implementing practical approaches such as inventory management techniques. In addition, there is a lack of proper policies for perishable inventory at food bank sectors, and future researchers can benefit from the optimization and DP methods to establish models for better inventory management of the perishable food banks. Further research is also required to evaluate better various OR methods to tackle pandemics and other disasters. With COVID-19 disruption, one can consider new source of uncertainty, addition to supplies and labour capacity, in various component of food bank operations such as client demands and distribution time. Addressing these new source of uncertainties can be considered new avenues for future direction of research. Traditional approaches such as SO and RO techniques can also be used to eradicate the problem more realistic.

## Data Availability

All data generated or analysed during this study are included in the published articles.
